# Optimization of Lead and Diclofenac Removal from Aqueous Media Using a Composite Sorbent of Silica Core and Polyelectrolyte Coacervate Shell

**DOI:** 10.3390/polym15081948

**Published:** 2023-04-19

**Authors:** Irina Morosanu, Florin Bucatariu, Daniela Fighir, Carmen Paduraru, Marcela Mihai, Carmen Teodosiu

**Affiliations:** 1 Department of Environmental Engineering and Management, Gheorghe Asachi Technical University of Iasi, 73 D. Mangeron Street, 700050 Iasi, Romania; morosanu.irina@gmail.com (I.M.); fbucatariu@icmpp.ro (F.B.); daniela.arsene@ch.tuiasi.ro (D.F.); cpadur2005@yahoo.com (C.P.); 2 Petru Poni Institute of Macromolecular Chemistry, 41A Grigore Ghica Voda Alley, 700487 Iasi, Romania

**Keywords:** silica, poly(ethyleneimine), diclofenac sodium salt, lead, sorption, optimization

## Abstract

The modification of inorganic surfaces with weak cationic polyelectrolytes by direct deposition through precipitation is a fast approach to generating composites with high numbers of functional groups. The core/shell composites present very good sorption capacity for heavy metal ions and negatively charged organic molecules from aqueous media. The sorbed amount of lead ions, used as a model for priority pollutants such as heavy metals, and diclofenac sodium salt, as an organic contaminant model for emerging pollutants, depended strongly on the organic content of the composite and less on the nature of contaminants, due to the different retention mechanisms (complexation vs. electrostatics/hydrophobics). Two experimental approaches were considered: (i) simultaneous adsorption of the two pollutants from a binary mixture and (ii) the sequential retention of each pollutant from monocomponent solutions. The simultaneous adsorption also considered process optimization by using the central composite design methodology to study the univariate effects of contact time and initial solution acidity with the purpose of enabling further practical applications in water/wastewater treatment. Sorbent regeneration after multiple sorption-desorption cycles was also investigated to assess its feasibility. Based on different non-linear regressions, the fitting of four isotherms (Langmuir, Freundlich, Hill, and Redlich–Peterson models) and three kinetics models (pseudo-first order (PFO), pseudo-second order (PSO), and two-compartment first order (TC)) has been carried out. The best agreement with experiments was found for the Langmuir isotherm and the PFO kinetic model. Silica/polyelectrolytes with a high number of functional groups may be considered efficient and versatile sorbents that can be used in wastewater treatment processes.

## 1. Introduction

Different statistical experimental design methods, e.g., the central composite design or the Box–Behnken design, were applied for investigating the simultaneous effect of several operating conditions on the adsorption efficiency with a minimum number of experiments [1,2]. Hiew et al. [3] studied the kinetics, equilibrium, and thermodynamics of sodium diclofenac (DCF-Na) sorption onto graphene oxide. The single and interaction effects of different parameters, such as sorbent dosage, contact time, initial concentration, and temperature, were analyzed by central composite design (CCD). Masoumi et al. [4] reported four process factors, namely temperature, pH, and initial concentrations of lead, nickel, and cadmium, respectively, in which the heavy metals adsorption was optimized by the CCD method of the response surface methodology (RSM). Optimization of DCF-Na sorption on eucalyptus wood biochar was carried out using the Box–Behnken design, considering the following variables: stirring rate, drug concentration, and sorbent dosage [5]. The interaction impact of various parameters, i.e., DCF-Na concentration, initial pH, sorbent dose, temperature, and contact time, was evaluated by the CCD of RSM for the prediction of the response and optimization of DCF-Na sorption on graphene oxide decorated with a zeolitic imidazolate framework [6]. A central composite design was preferred among other RSM techniques for the optimization of DCF-Na sorption on a quaternized mesoporous silica, considering initial pH, sorbent dosage, reaction time, and initial concentration [7].

In our previous study [8], the composite sorbent obtained by direct deposition on an inorganic silica core (IS) of a coacervate based on poly(ethyleneimine) (PEI)/poly(acrylic acid) (PAA) and PEI/poly(sodium methacrylate) (PMAA), which underwent a strong crosslinking, presented the highest sorption capacity for Cd^2+^ ions as compared to layer-by-layer polyelectrolyte composite sorbents. This paper focused on the potential of the same composite sorbent, obtained by direct deposition of a coacervate, to remove lead ions (Pb^2+^) and DCF-Na from aqueous solutions. Two main approaches were considered: (i) simultaneous adsorption of the two pollutants from a binary mixture and (ii) the alternative retention of each pollutant from monocomponent solutions. The first approach considered the optimization of the sorption process and the univariate effects of contact time and initial solution acidity. In this sense, a popular response surface experimental design based on CCD was used to model the simultaneous adsorption of Pb^2+^ ions and DCF-Na from aqueous solutions. The novelty of this paper lies in reporting the use of the CCD methodology for the optimization of the operating conditions for the simultaneous adsorption of these two priority pollutants. It is of high interest to determine the impact of different ratios between the two pollutants in solution by experimental design and to optimize the operational process conditions. Therefore, the experimental design was composed of twenty batch experimental runs, which were used to evaluate the influence of important operating parameters and their interactions upon the bicomponent adsorption system. Sorbent regeneration after multiple sorption-desorption cycles was also investigated to assess the feasibility of the sorbent. The second approach is sequential adsorption, with the focus on the possibility of functionalizing the sorbent with metal ions for improved DCF-Na sorption.

## 2. Materials and Methodology

### 2.1. Materials

All chemical reagents, i.e., lead nitrate (Fluka, Buchs, Switzerland), diclofenac sodium salt (98%, Acros Organics, Geel, Belgium), methanol Uvasol (Merck, Bucuresti, Romania), hydrochloric acid (Merck, Bucuresti, Romania), sodium hydroxide (Merck, Bucuresti, Romania), and ethylenediaminetetraacetic acid disodium salt (EDTA, Merck, Bucuresti, Romania), were of analytical purity and used as received. Silica particles of 40–60 microns in diameter were purchased from Daiso Co. (Osaka, Japan). Poly(sodium methacrylate) (M_w_ = 1800 g/mol) and branched poly(ethyleneimine) (M_w_ = 25,000 g/mol) were acquired from Aldrich (Redox, Otopeni, Romania) and used as is.

IS/(PEI/PMAA)_c_ composite sorbent was prepared as described in our previous study [8]. Briefly, 5.0 g of silica microparticles (spherical shape) were first dispersed into 1 mol L^−1^ PEI (12 mL), and then the addition of 1 mol L^−1^ PMAA (6 mL) was made dropwise under vigorous stirring with a glass rod until a transparent solution was obtained. Then, PEI crosslinking with glutaraldehyde (2.5% *w*/*v*) was performed, obtaining a crosslinking ratio of [−CHO]:[−NH_2_] = 1:1. Finally, the core/shell composite microparticles were treated with 1 mol L^−1^ of NaOH for PMMA extraction.

### 2.2. Batch Sorption and Desorption Studies

The preliminary investigation consisted of verifying the influence of solution acidity (4 < pH < 6) and contact time (1–8 h) on the simultaneous sorption of Pb^2+^ ions and DCF-Na on the IS/(PEI/PMAA)_c_ composite sorbent. The pH of the solution was adjusted with 0.01 N HNO_3_. A sorption equilibrium was considered to be attained when the sorption capacities of two successive samples did not vary significantly with time. The effect of the pollutants initial concentrations was studied considering the concentration ranges from the experimental design. All univariate experiments were conducted utilizing 2 g of composite per liter of aqueous solution, at room temperature.

The DCF-Na concentration was determined by a direct spectrophotometric method at a λ_max_ = 276 nm using Analytik Jena Specord 210 Plus (Analytik-Jena, Bucuresti, Romania) equipment. The concentration of Pb^2+^ ions in water solutions was quantified using an Analytik Jena 800 atomic absorption spectrometer (Analytik-Jena, Bucuresti, Romania). The calibration curves were validated by a certified reference material, namely diclofenac sodium 1 mg/mL in methanol from LGC and a multi-element metal standard solution from Merck. In addition, the analysis methods were verified for any interference from other components of the binary mixture. For a lead concentration of up to 71 mg/L, no interference could be detected on the DCF-Na determination. Additionally, no interference was observed for DCF-Na up to 65 mg/L in the analysis of lead ions. 

The mass of Pb^2+^ ions or DCF-Na retained on IS/(PEI/PMAA)_c_ (r = 1.0) was evaluated from the mass balance of the pollutant, which is represented by the following equation:

(1) $$q_{i}=\left(C_{0}-C_{i}\right)V/m$$

where *q_i_* (mg/g polymer) is the sorption capacity at a certain time *t* (*q_t_*) or at equilibrium (*q_e_*), *C*_0_ (mg/L) is the initial concentration, *C_i_* (mg/L) is the concentration at a certain time *t* (*C_t_*) or at equilibrium (*C_e_*), *m* (g) is the sorbent amount, and *V* (L) is the volume of the solution.

The pollutant removal efficiency (*RE*, %) was calculated as follows:

(2) $$RE=\left(C_{0}-C_{i}\right)\;100/C_{0}.$$

The regeneration of the composite sorbent was achieved by repeatedly washing the material with EDTA (0.1 mol/L) and HCl (1 mol/L), followed by activation with NaOH (1 mol/L) [8].

The sequential sorption tests were carried out by contacting the composite sorbent with one mono-element solution of each of the pollutants at a time [9] in order to investigate the possibility of sorbent functionalization and sorption efficiency.

### 2.3. Adsorption Kinetics and Isotherm Models

The sorption data obtained at different contact times have been described by pseudo-first order, pseudo-second order, two-compartment first-order kinetics models, as well as a liquid film diffusion equation. The sorption process was investigated through the following equilibrium isotherms: Langmuir, Freundlich, Hill, and Redlich–Peterson models. 

The following objective functions were minimized in turn, and the optimum model parameters were chosen by the minimum sum of normalized errors:

(3) $$\mathrm{sum}\;\mathrm{of}\;\mathrm{the}\;\mathrm{square}\;\mathrm{error}\;(\mathrm{SSE}),\;\sum_{j=1}^{x}{\left(q_{j,exp}-q_{j,model}\right)}^{2},$$

(4) $$\mathrm{chi}-\mathrm{square}\;\mathrm{test}\;(\chi^{2}),\;\frac{\sum_{j=1}^{x}{\left(q_{j,exp}-q_{j,model}\right)}^{2}}{q_{i,exp}},$$

(5) $$\mathrm{average}\;\mathrm{relative}\;\mathrm{error}\;(\mathrm{ARE}),\;\frac{100}{x}\sum_{j=1}^{x}\left|\frac{q_{j,exp}-q_{j,model}}{q_{j,exp}}\right|.$$

The best-fit model was selected based on the smallest value of the Hannan–Quinn information criterion [10].

### 2.4. Design of Experiments

The influence of the initial priority pollutant concentrations and the adsorption temperature were studied using the central composite design along with the coded independent variables shown in Table 1. The experimental design was composed of six center points, six axial points, and eight factorial points, which were obtained using Design Expert software, version 10.0.1 (DX10, Stat-Ease, US). The design matrix is indicated in Table 2, along with the two response functions considered—the sorption capacity for each of the priority pollutants involved and their predicted values.

The behavior of the simultaneous adsorption of Pb^2+^ ions and DCF-Na on the IS/(PEI/PMAA)_c_ (r = 1.0) composite sorbent was mathematically modeled by a second-order polynomial equation (Equation (6)), which considers the interaction between the independent process variables:

(6) $$q_{pred}=\beta_{0}+\sum_{i=1}^{n}\beta_{i}x_{i}+\sum_{i=1}^{n}\beta_{ii}x_{i}^{2}+\sum_{i=1}^{n-1}\sum_{j=i+1}^{n}\beta_{ij}x_{i}x_{j}+\varepsilon ,$$

where *q_pred_* is the predicted sorption capacity (mg/g polymer), *x_i_*, *x_i_*^2^, and *x_i_x_j_* are the individual variables, the quadratic effects, and the interactions between variables, respectively; *β*_0_ is a constant; *β_i_*, *β_ii_*_,_ and *β_ij_* are the linear, quadratic, and interaction coefficients, respectively; and *ε* is the random error. The total number of variables *n* = 3.

The regressed models were optimized by the analysis of variance (ANOVA) with the DX10 software. ANOVA indicated the statistical significance and influence of the independent model parameters and their interactions. For this purpose, a probability value (*p*-value), the Fischer’s test value (*F*-value) at a 95% confidence level, the coefficient of determination (*R*^2^), adjusted *R*^2^ (*R*^2^*_adj_*), and predicted *R*^2^ (*R*^2^*_pred_*), as well as normalized residue plots, were used.

## 3. Results and Discussion

### 3.1. Univariate Simultaneous Experiments

**Figure 1:**
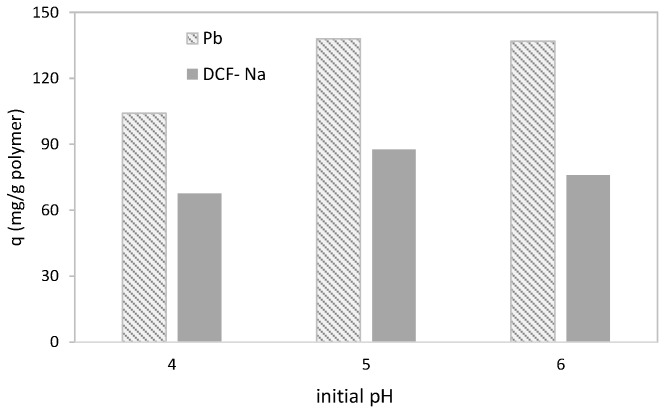
Effect of solution acidity on the simultaneous sorption process.

The influence of solution acidity on the efficiency of the simultaneous sorption process is illustrated in Figure 1. The experiments were carried out at an initial concentration of 47 mg of Pb^2+^/L and 30 mg of DCF-Na/L. The sorption capacity of both pollutants increases with an increase in the initial pH. The highest values of the sorption capacity were obtained at a pH of 5, reaching a *q_Pb_* = 138 mg/g polymer and a *q_DCF-Na_* = 87.6 mg/g polymer. Hence, the optimum pH was considered 5, and the subsequent tests were performed using this value.

At pH = 5, near the pK_a_ of DCF-Na (~4.2) and the point of zero charge of the sorbent (~4.3), the DCF-Na molecules with the carboxylic groups interacted electrostatically with protonated amino groups inside the composite shell. Moreover, π-stacking, hydrogen bonds, and hydrophobics could favor DCF-Na retention as possible secondary forces inside the shell. The Pb^2+^ ions interacted, inside the cross-linked shell, with the amino groups of the PEI by forming coordinative bonds. Due to different types of pollutant entity interactions, coordinative for Pb^2+^ and electrostatic/H-bonds/hydrophobics for DCF-Na, we can conclude that the sorption could be additive at the same sorption site inside the cross-linked organic part of the composite. Thus, the DCF-Na sorbed amount is not influenced drastically by the Pb^2+^ sorbed amount. DCF-Na can form a complex with lead ions (molar ratio 2:1) [11] and further interact with Pb^2+^ as a counter ion, replacing nitrate ions. 

**Figure 2:**
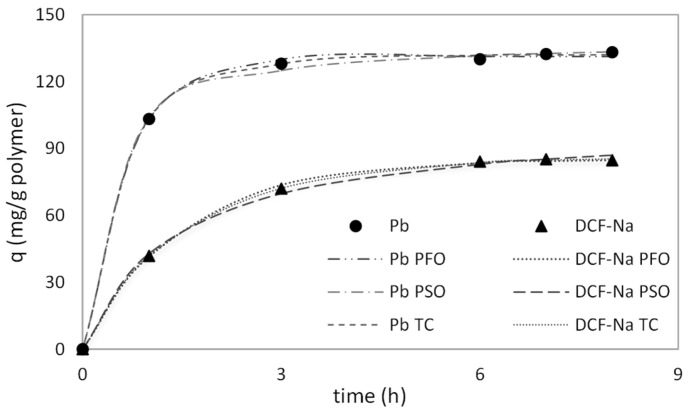
The influence of contact time on the sorption capacity of IS/(PEI/PMAA)_c_ (r = 1.0) on the lead ions and diclofenac sodium from a bi-component mixture.

The variation with time of the sorption capacity for the two pollutants, illustrated in Figure 2, shows that the sorption process occurs in two stages. The first stage is characterized by the rapid migration of the pollutants from the liquid phase to the sorbent’s surface. For lead ions, this happens in the first hour of contact between the solid and liquid phases, when a sorption capacity of 103.3 mg/g polymer is achieved. After 3 h, the increment in the sorption capacity is small because the process advances slowly. For the organic pollutant, the initial adsorption stage takes place after around 2 h and after 6 h, equilibrium is reached. Due to the slower sorption kinetics of the sodium diclofenac, the optimum contact time between the binary mixture and IS/(PEI/PMAA)_c_ (r = 1.0) was considered to be 6 h. Hence, all experiments, including the CCD tests, were performed within this time interval.

The kinetics parameters for the pseudo-first order (PFO) [12], pseudo-second order (PSO), and two-compartment first-order (TC) [13] models are presented in Table 3. The PFO equation assumes that the sorption rate is proportional to the distance from equilibrium, whereas the PSO equation describes that the rate is proportional to the second power of the distance from equilibrium [14].

Analyzing the results for the kinetics of lead ions sorption displayed in Table 1, it can be observed that the error functions of the PFO and PSO models show close values for *R*^2^, *χ*^2^, and *ARE*, but lesser values for *SSE*, and hence, for the *HQ* information criterion. In the case of DCF-Na sorption kinetics, this is noticeable for PFO and the two-compartment model. According to Azizian [15], a high initial concentration of solute determines a better fit with the pseudo-first-order kinetics, while the sorption kinetics follows the PSO model better when the initial concentration is not too high. The experimental equilibrium sorption capacity was 133.2 mg/g for the Pb^2+^ ions and 84.7 mg/g for the DCF-Na. All models estimated *q_e, Pb_* values close to the experimental ones, but the estimation of *q_e, DCF-Na_* for the PSO model was 20% higher than the experimental one. It was reported that sorption kinetics often follow the PFO equation when the process occurs through diffusion through an interface [16].

Based on the smaller values for the HQ criterion, the degree of goodness-of-fit decreases in the following order: two-compartment, PFO, and then PSO for both pollutants. The TC model assumes that the sorption process takes place as a two-phase process [13]. The TC equation provided the best fit with the experimental data, giving the best estimations of the experimental sorption capacities on IS/(PEI/PMAA)_c_ (r = 1.0). The constants mass fraction *F_fast_* and first-order rate *k_fast_*, corresponding to the rapid initial sorption stage, are larger than those of the slow compartment, suggesting that the fast sorption stage predominated during the pollutants uptake by the composite sorbent.

**Figure 3:**
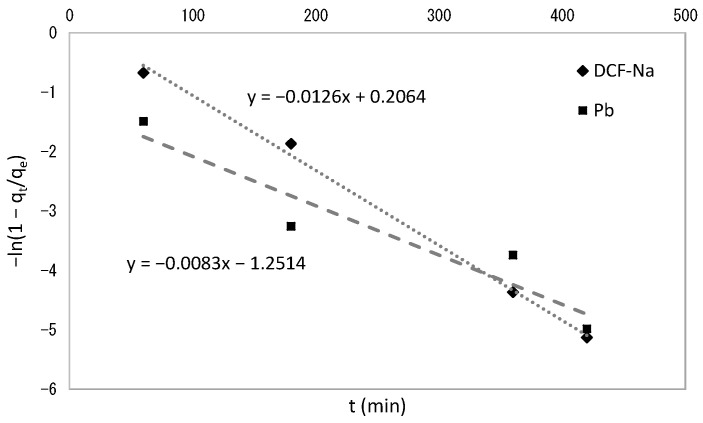
Film diffusion plot for the simultaneous sorption process.

**Figure 4:**
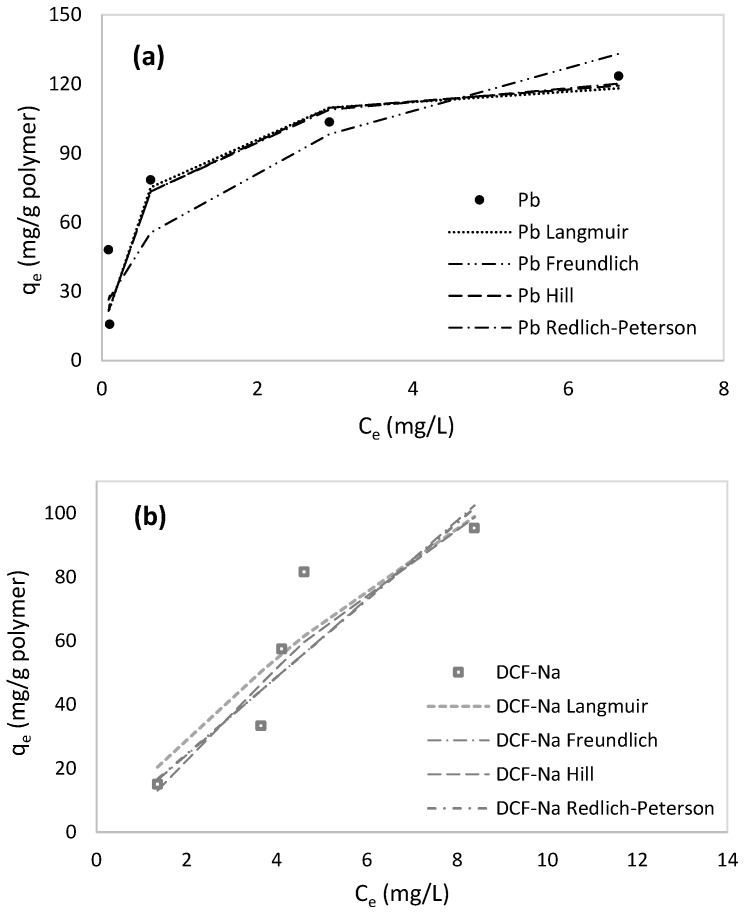
Isotherms of lead (**a**) and diclofenac sodium (**b**) for simultaneous sorption process.

The diffusion mechanism was analyzed by applying the film diffusion mass transfer rate model [17]. A linear plot of –ln(1 – *q_t_*/*q_e_*) vs. *t* with an intercept of zero indicates that the sorption kinetics are governed by the diffusion through the liquid film surrounding the sorbent microparticles. The plots depicted in Figure 3 show a deviation from linearity for lead ion sorption, while the curve for DCF-Na presents very good linearity, with an intercept very close to zero. When the line for DCF-Na is forced to go through the origin, the coefficient *R*^2^ becomes 0.9925. Hence, it can be considered that the curve for DCF-Na meets the conditions for the film diffusion to be rate-controlled. On the other hand, diffusion through the liquid film is not the main determining step in the kinetics of lead ion uptake by IS/(PEI/PMAA)_c_ (r = 1.0). The sorption isotherms of lead (Figure 4a) and diclofenac sodium (Figure 4b) are presented in Figure 4.

Relevant parameters of the four isotherm equations, i.e., Langmuir [18], Freundlich [19], Hill [20], and Redlich–Peterson [21], are presented in Table 4. According to the HQ information criterion, the Langmuir model describes best the uptake of the pollutants on the composite sorbent, followed by Redlich–Peterson, Hill, and Freundlich for Pb^2+^ ion sorption and by Hill, Freundlich, and Redlich–Peterson for DCF-Na sorption.

The Langmuir model describes the equilibrium condition of a monolayer coverage of a homogeneous surface having identical adsorption sites [18]. Saturation sorption capacities of 125.68 mg/g polymer and 388.35 mg/g polymer were determined for Pb^2+^ and DCF-Na, respectively. A larger *K_L_* equilibrium coefficient for lead ions suggests a stronger affinity of the sorbent towards this contaminant. In addition, the Langmuir isotherm and HQC indicate that the Redlich–Peterson and Hill models also describe the Pb^2+^ ion equilibrium data fairly well. The Redlich–Peterson model is an empirical isotherm, incorporating both the Langmuir and Freundlich models. As such, it presents a constant (*K_RP_*) quantifying the linear affinity of concentration in the numerator and an exponent in the denominator to explain sorption saturation over a wide range of concentrations, providing the opportunity to apply the model to homogeneous and heterogeneous sorption processes [4]. The Hill isotherm model can explain the sorption of contaminants as a cooperative binding process, where the pollutant species bind to the active sites on a homogeneous substrate, producing an effect on the binding at other sites in the vicinity [22]. As noticed in Table 4, both isotherm exponents, *β* and *n_H_*, are close to unity and thus reduce the models to the Langmuir equation. In this case, the closeness of the maximum sorption capacity between the three models is verified: for Langmuir—125.68 mg/g polymer; for Hill—129.11 mg/g polymer; and for Redlich–Peterson—*q_m_* ≈ *K_RP_*/*α* = 118.51 mg/g polymer. 

In the case of DCF-Na sorption on the composite surface, the highest *R*^2^ was 0.837 for the Langmuir isotherm, indicating that at most 83.7% of the data can be explained by this model. The maximum sorption capacity indicated by this model is 388.35 mg/g polymer, which is higher than the DCF-Na individual sorption of 128.01 mg/g polymer (Hill model, *R*^2^ = 0.996, data not shown). This could be explained by the overestimation of the Langmuir isotherm in the case of simultaneous sorption due to the enhancement of DCF-Na sorption by Pb^2+^. This fact could be attributed to the ionic exchange of the NO_3_^−^ ion being replaced by the carboxylate group of ionized DCF-Na. 

A comparison of the maximum sorption capacity of the IS/(PEI/PMAA)_c_ (r = 1.0) sorbent with other materials reported for the sorption of lead ions and/or diclofenac sodium is presented in Table 5.

### 3.2. Multivariate/Multi-Objective Simultaneous Process Modeling and Optimization

#### 3.2.1. Statistical Analysis of the Process Model

The experimental data from Table 2 were subjected to multiple regression analysis to fit the quadratic model (Equation (6)) for the two responses: *Y*_1_—the sorption capacity of lead ions (*q_Pb_*) and *Y*_2_—the sorption capacity of diclofenac sodium (*q_DCF-Na_*). The mathematical expressions, in actual units, are given below:

(7) $$q_{Pb}=-6.65936+3.90003C_{0,Pb}+0.038835C_{0,DCF-Na}+0.16015T\;+0.007723C_{0,Pb}C_{0,DCF-Na}-0.010766C_{0,Pb}T+0.0004017C_{0,DCF-Na}T\;-0.023225C_{0,Pb}^{2}-0.0040925C_{0,DCF-Na}^{2}+0.0012756T^{2},$$

(8) $$q_{DCF-Na}=-5.67172+0.18502C_{0,Pb}+2.57842C_{0,DCF-Na}+0.076041T.$$

The goodness-of-fit and lack-of-fit test results obtained by ANOVA are presented in Table 6. A second-order polynomial model was developed to predict the influence of parameters on lead ion sorption on the IS/(PEI/PMAA)_c_ (r = 1.0) composite sorbent, while a linear model was obtained for diclofenac sodium sorption. In the latter, no interaction between the parameters could be observed. As seen in Table 6, both models are statistically significant based on a determined *p*-value of less than 0.0001 and high values for the *F*-value. The linear and quadratic effects of the initial concentration of metallic ions were significant on the sorption behavior of lead ions (*p*-value < 0.05). The sorption capacity of DCF-Na is significantly impacted (*p*-value < 0.05) by the initial concentration of both organic and inorganic pollutants in the binary system. The lack-of-fit test indicates if the model is adequate by measuring the error arising from a deficiency in the model: if the *F*-value of the lack-of-fit error is large and the corresponding error probability is small, the model is a poor fit to the experimental data [33]. The ANOVA summary shows the sorption of the two pollutants in this paper as a non-significant lack-of-fit for the regression models. The lack-of-fit *F*-values of 2.63 and 2.87, respectively, suggest that the lack-of-fit error is not significant in comparison with the pure error. 

The goodness-of-fit test, given by the *R^2^* coefficient, showed that over 99% of the variation in the observed data is explained by the generated models. In both cases, the predicted *R^2^* was in agreement with the adjusted *R*^2^, with a difference of less than 0.2. This shows a good correlation between the experimental and predicted sorption capacities. The signal-to-noise ratio, expressed by the adequate precision value, should be higher than four for a good response signal and for the model to be suitable for use, as can be seen in Table 6. 

**Figure 5:**
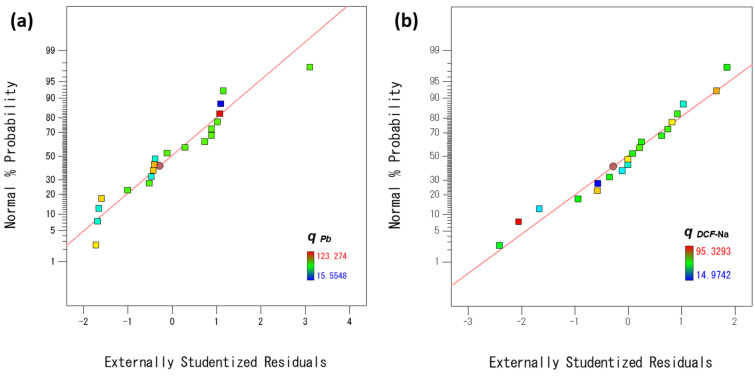
Normal plots of residuals for (**a**) *q_Pb_* and (**b**) *q_DCF-Na_*.

**Figure 6:**
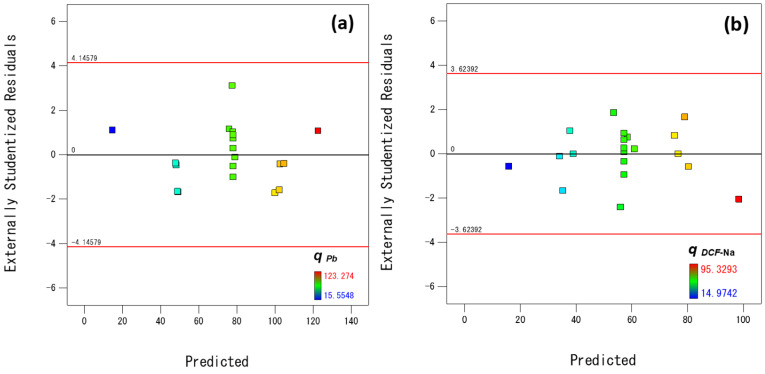
Diagnostic plots of residuals versus predicted responses for (**a**) *q_Pb_* and (**b**) *q_DCF-Na_*.

**Figure 7:**
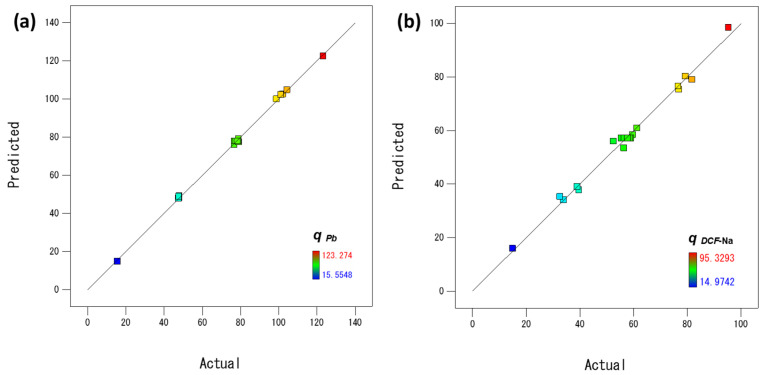
Plots of experimental versus predicted responses for *q_Pb_* (**a**) and *q_DCF-Na_* (**b**).

The models’ adequacy was further analyzed by diagnostic plots. The plots of the normal distribution of studentized residuals shown in Figure 5 are characterized by good linearity, with no large variation from the straight line for both responses, *q_Pb_* and *q_DCF-Na_*. To check the constant error, the plot of the residuals relative to the predicted responses was used. A uniform distribution of the residuals, as depicted in Figure 6, suggests that the variance is constant. Finally, the accuracy of the models is confirmed by the closeness between the experimental sorption capacities and the values obtained from the models, as seen in Figure 7 and Table 2.

Based on the results of the analysis of variance and the diagnostic plots, the developed models could be used to describe the sorption of Pb^2+^ ions and DCF-Na on IS/(PEI/PMAA)_c_ (r = 1.0) at a 95% confidence level.

#### 3.2.2. Effects of Factors

The degree of influence and the corresponding effect of each independent variable on the process responses, *q_Pb_* and *q_DCF-Na_*, can be determined from the sign and values of the models’ coefficients, as shown in Table 7. The negative sign of temperature, the *C_0,Pb_*-*T* interaction, and the quadratic effect of the pollutant’s initial concentration indicated the negative impact on the Pb^2+^ ions’ sorption capacity. On the other hand, all parameters presented a positive influence on DCF-Na sorption, as the coefficients’ signs were positive. The magnitudes of *C_0,Pb_* and *C_0,DCF-Na_* coefficients were higher than the other terms, suggesting these variables have more influence on the process responses.

**Figure 8:**
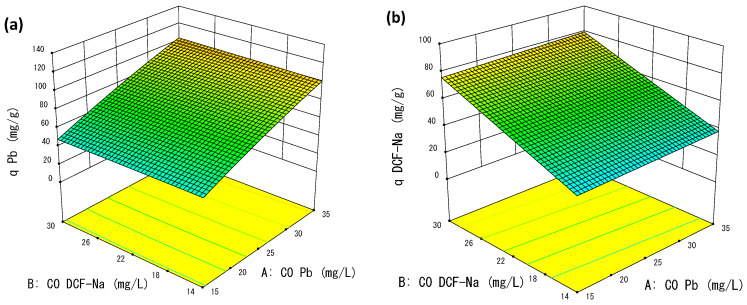
Three-dimensional response surface of the effect of lead ions and DCF-Na initial concentrations (at a temperature of 20 °C) on the simultaneous sorption process: (**a**) *q_Pb_* and (**b**) *q_DCF-Na_*.

One aspect of this study is to explore the effect of the pollutant initial concentration on the lead and DCF-Na simultaneous adsorption from aqueous media. As seen until now, the CCD method was useful in determining if an interaction between these variables existed or not. Moreover, the 3D response surface plots were used to examine the interaction between the two variables, while the other factors were maintained at fixed values. The interaction between *C_0,Pb_* and *C_0,DCF-Na_* on the sorption capacity *q_Pb_* is illustrated in Figure 8a. The response *q_Pb_* varies between 15.6 and 123.3 mg/g polymer and increases with the initial concentration of Pb^2+^ ions due to a higher driving force between the liquid phase and the sorbent surface. The presence of DCF-Na with an initial concentration between 14 and 30 mg/L does not affect the metal ion sorption from the binary mixture. Looking back at the experimental results, at Pb:DCF-Na molar ratios between 1.0 and 6.41 (C_0,Pb_ = 25 mg/L), the capacity *q_Pb_* only varies slightly (about 1.2%). 

In the current study, the initial concentration of DCF-Na varied from 6 to 38 mg/L. An increase from 15 to 95.3 mg/g polymer in the *q_DCF-Na_* values was noticed with an increase in the organic moiety in the binary sorption system. A closer inspection of Figure 8b shows a small increase in *q_DCF-Na_* at a high concentration of lead ions. Indeed, the experimental data indicates an augmentation of 8% in the sorption capacity of DCF-Na from a Pb:DCF-Na molar ratio of 0.34–3.14 (*C_0,DCF-Na_* = 22 mg/L). 

The third factor considered in this study, e.g., the adsorption temperature, was found to have a weak influence on both lead ions and DCF-Na sorption from the binary mixture. This proves that the IS/(PEI/PMAA)_c_ (r = 1.0) composite sorbent is not a thermosensitive material for ionic pollutants separation from aqueous media.

#### 3.2.3. Optimization of the Adsorption Process

The sorption of Pb^2+^ ions and DCF-Na from binary mixtures on IS/(PEI/PMAA)_c_ (r = 1.0) is optimized by the desirability function based on the pollutants sorption capacities as a response. The highest value of the desirability function was obtained for the following conditions: *C_0,Pb_* = 35 mg/L, *C_0,DCF-Na_* = 30 mg/L, and *T* = 12 °C, when the sorption capacities were *q_Pb_* = 104.71 mg/g polymer and *q_DCF-Na_* = 79.069 mg/g polymer. Sorption tests were performed in quadruplicate according to the mentioned conditions, and the experimental results indicated a deviation of −2.27% and 0.23% from the optimized *q* values for Pb^2+^ ions and DCF-Na, respectively. At a temperature of 20 °C, for the same initial pollutant concentrations (a desirability of 0.810), the experimental values deviated by −1.81% and 2.76% for *q_Pb_* and *q_DCF-Na_*, respectively, as against the optimized responses. These results demonstrate the accuracy of the developed models.

### 3.3. Desorption and Sorbent Regeneration

**Figure 9:**
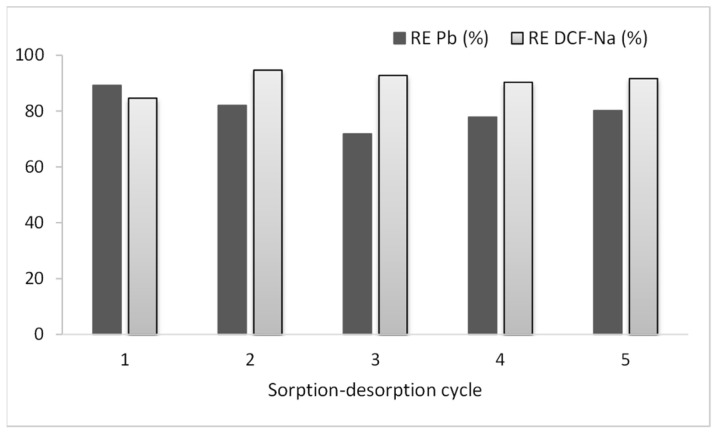
Removal efficiency (RE) of the pollutants in consecutive adsorption-desorption cycles.

Experiments of pollutants’ desorption were carried out at room temperature, with initial optimized concentrations from the RSM-CCD design of experiments. The simultaneous sorption efficiency for the two contaminants is presented in Figure 9. The removal efficiency (RE) of Pb^2+^ ions varied from a high of 89.2% to a low of 72%, while for DCF-Na the RE remained almost constant after five cycles of sorption-desorption.

### 3.4. Sequential Sorption

**Figure 10:**
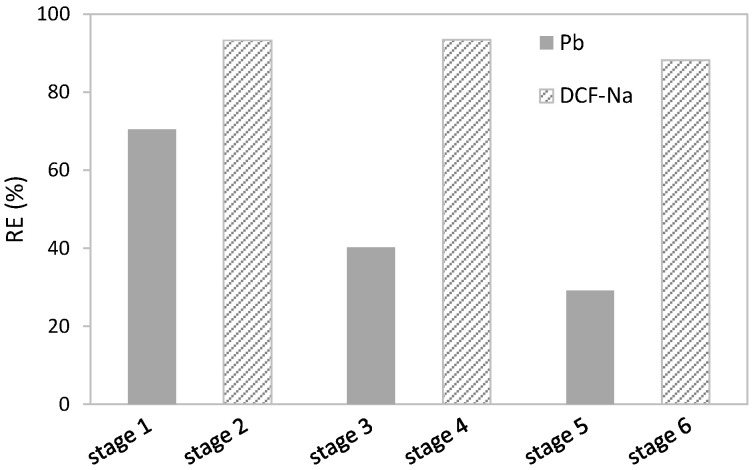
DCF-Na and Pb^2+^ ion removal efficiencies in sequential sorption experiments.

The sequential sorption experiments were carried out at room temperature for 7 h, with *C_0,Pb_* = 43 mg/L and *C_0,DCF-Na_* = 30 mg/L. The results, presented in Figure 10, indicate a significant decrease of lead ions *RE* from 70.4% to 29.2%. 

This fact may be explained by the saturation of the sorbent surface as the available active sites, especially amino groups, for dative bond formation decrease. Meanwhile, a high DCF-Na is maintained even in stage six, when it reaches a value of 88.2%. At the same time, a leaching of Pb^2+^ ions was observed experimentally in stages four (16%) and six (20.9%), possibly due to the formation of Pb(DCF)_2_ in solution by ionic exchanges. The physico-chemical interactions are tremendously important because they dictate the sorbent capacity for pollutant detection, retention, concentration in the solid phase, and subsequent release in the desorption step. The processes of retention of inorganic (Pb^2+^) or organic (DCF-Na) pollutants are driven by coordinative bond formation, electrostatic interactions, H-bonding, hydrophobic interactions, or ionic exchange interactions.

**Scheme 1:**
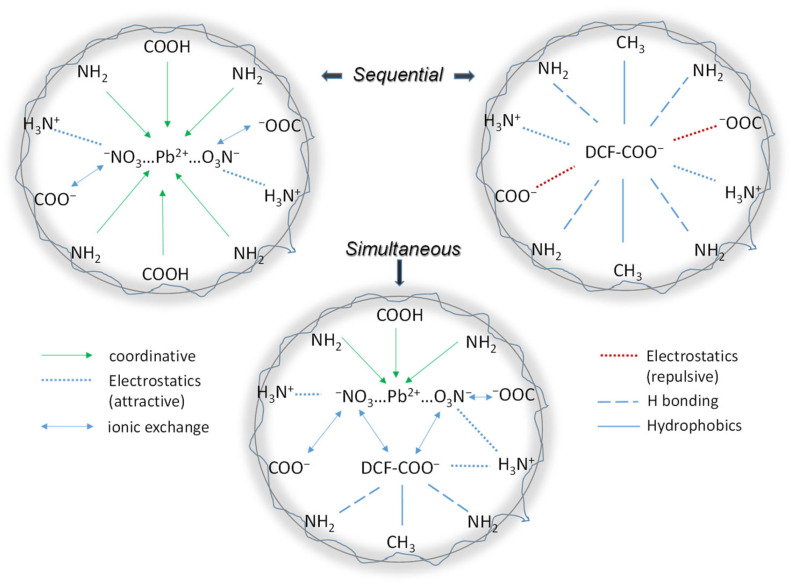
Different types of interactions between Pb^2+^ and DCF-Na with composites based on PEI/PMAA chains on the surface.

The schematic representation of possible interactions between pollutants and the composite surface, presented in Scheme 1, gave a clear overview of the advantages of using core/shell composite particles with different functional groups on the surface, which can act as active sites for the removal by sorption of different types of pollutants.

## 4. Conclusions

This study proposed to investigate the sorption processes in batch conditions of two model pollutants, Pb^2+^ and DCF-Na, onto a core/shell composite based on PEI and PMAA. The synthesis strategy of the sorbent has been previously reported; in this approach, only one support (IS/(PEI/PMAA)_c_), which contains the highest deposited amount of polyelectrolytes (~20% organic), has been tested as a sorbent. The sequential and simultaneous sorption cycles of lead ions and DCF-Na molecules, carried out in batch conditions, showed an additive capacity of the composite towards two different types of pollutants, with an important amount of DCF-Na being sorbed together with Pb^2+^ ions. This additive sorption demonstrated that the chemical (coordinative bonds) and physical (electrostatic, H-bonding, and hydrophobic) interactions are the main driving forces for pollutants retention at the same active sorption sites inside the cross-linked organic shell. The process optimization used the central composite design to model the simultaneous adsorption of Pb^2+^ ions and DCF-Na from aqueous solutions for various operating conditions. The experimental design was composed of twenty batch experimental runs and was used to evaluate the impact of different ratios between the two priority pollutants in solution and find optimum conditions for simultaneous adsorption. This study proved that this type of composite, obtained by the direct deposition of polyelectrolytes, may be used for the removal from wastewater of two main classes of priority pollutants: heavy metals and anionic organic molecules.

## Figures and Tables

**Table 1:** Experimental design levels of factors for Pb^2+^ ions and DCF-Na simultaneous adsorption on IS/(PEI/PMAA)_c_ (r = 1.0).

Factor	Units	Level				
		−2	−1	0	+1	+2
A: C_0 Pb_	mg/L	5	15	25	35	45
B: C_0 DCF-Na_	mg/L	6	14	22	30	38
C: T	°C	4	12	20	28	36

**Table 2:** CCD matrix with experimental (*exp*) and predicted (*pred*) responses.

Run	A	B	C	*Y*1: *q_exp, Pb_* (mg/g Polymer)	*q_pred, Pb_* (mg/g Polymer)	Residual (*q_exp, Pb_* − *q_pred, Pb_*)	*Y*2: *q_exp, DCF-Na_* (mg/g Polymer)	*q_pred, DCF-Na_* (mg/g Polymer)	Residual (*q_exp, DCF-Na_* − *q_pred, DCF-Na_*)
1	35	30	12	104.39	104.71	−0.32	81.80	79.07	2.73
2	25	22	20	78.98	77.95	1.02	58.44	57.20	1.24
3	45	22	20	123.27	122.53	0.74	61.27	60.90	0.37
4	35	14	12	102.23	102.57	−0.34	39.60	37.81	1.79
5	25	22	20	77.35	77.95	−0.60	57.36	57.20	0.16
6	25	22	4	78.98	79.07	−0.084	52.46	55.98	−3.52
7	5	22	20	15.55	14.80	0.76	56.37	53.50	2.87
8	25	22	20	76.82	77.95	−1.13	55.37	57.20	−1.83
9	35	14	28	98.81	100.01	−1.20	39.02	39.03	−0.011
10	25	22	36	79.08	77.49	1.59	59.67	58.42	1.26
11	15	30	12	47.58	47.88	−0.30	76.81	75.37	1.44
12	15	14	28	47.92	49.10	−1.18	32.58	35.33	−2.75
13	35	30	28	101.12	102.25	−1.13	79.25	80.29	−1.03
14	25	22	20	78.81	77.95	0.85	56.50	57.20	−0.70
15	25	38	20	78.57	77.86	0.71	95.33	98.45	−3.13
16	25	22	20	78.30	77.95	0.34	57.70	57.20	0.50
17	15	14	12	47.84	48.21	−0.37	33.91	34.11	−0.21
18	25	22	20	78.98	77.95	1.02	59.00	57.20	1.80
19	25	6	20	76.74	75.95	0.79	14.97	15.95	−0.97
20	15	30	28	47.71	48.87	−1.17	76.58	76.59	−9.537 × 10^−3^

**Table 3:** Kinetics parameters for Pb^2+^ ions and DCF-Na simultaneous sorption on IS/(PEI/PMAA)_c_ (r = 1.0).

		Pb^2+^ Ions	DCF-Na
Pseudo-first order $q_{t}=q_{e,1}\left(1-\exp\left(-k_{1}t\right)\right)$	*q_e_* (mg/g)	131.27	85.18
	*k*_1_ (min^−1^)	0.02562	0.01112
	*R* ^2^	0.984	0.997
	*SSE*	9.82	3.72
	*χ* ^2^	0.08	0.05
	*ARE*	0.97	0.96
	*HQC*	5.28	0.43
Pseudo-second order $q_{t}=\frac{k_{2}q_{e,2}^{2}t}{1+k_{2}q_{e,2}t}$	*q_e_* (mg/g)	139.06	102.06
	*k*_1_ (min^−1^)	0.000356	0.000118
	*R* ^2^	0.980	0.991
	*SSE*	12.71	12.52
	*χ* ^2^	0.10	0.17
	*ARE*	0.96	1.89
	*HQC*	6.57	6.49
Two-compartment $q_{t}=q_{e}F_{fast}\left(1-e^{-tk_{fast}}\right)+q_{e}F_{slow}\left(1-e^{-tk_{slow}}\right)$, *F_fast_* + *F_slow_* = 1	*q_e_* (mg/g)	132.02	86.16
	*F_fast_*	0.425	0.084
	*k_fast_* (min^−1^)	0.247	0.212
	*F_slow_*	0.575	0.916
	*k_slow_* (min^−1^)	0.016	0.010
	*R* ^2^	0.993	0.999
	*SSE*	4.64	0.86
	*χ* ^2^	0.04	0.01
	*ARE*	0.53	0.41
	*HQC*	4.39	−4.06
Film diffusion $\ln\left(1-\frac{q_{t}}{q_{e}}\right)=-k_{fd}t$	*k_fd_* (min^−1^)	0.0083	0.0126
	*R* ^2^	0.899	0.996

**Table 4:** Isotherm parameters for the adsorption of DCF-Na and Pb^2+^ ions.

		Pb^2+^ Ions	DCF-Na
Langmuir $q_{e}=\frac{q_{m,L}K_{L}C_{e}}{1+K_{L}C_{e}}$	*q_m,L_* (mg/g)	125.68	388.35
	*K_L_* (L/mg)	2.36	0.04
	*R* ^2^	0.907	0.837
	*SSE*	868.38	739.89
	*χ* ^2^	20.31	15.79
	*ARE*	24.90	23.69
	*HQC*	27.69	26.89
Freundlich $q_{e}=K_{F}C_{e}^{\frac{1}{n_{F}}}$	*n_F_*	2.69	0.99
	*K_L_* (mg/g)(mg/L)^1/*n*^*_F_*	65.84	12.05
	*R* ^2^	0.871	0.810
	*SSE*	1262.84	878.98
	*χ* ^2^	27.29	13.25
	*ARE*	33.39	18.84
	*HQC*	29.56	27.75
Hill $q_{e}=\frac{q_{m,H}C_{e}{}^{n_{H}}}{K_{S}+C_{e}{}^{n_{H}}}$	*q_m,H_* (mg/g)	129.11	177.18
	*K_H_*	0.49	20.05
	*n_H_*	0.94	1.52
	*R* ^2^	0.909	0.849
	*SSE*	849.74	692.16
	*χ* ^2^	20.25	11.86
	*ARE*	25.39	18.16
	*HQC*	28.53	27.51
Redlich–Peterson $q_{e}=\frac{K_{RP}C_{e}}{1+\alpha C_{e}^{\beta }}$	*K_RP_* (L/g)	314.28	13.89
	*α* (L/mg)*^β^*	2.652	0.135
	*β*	0.96	0.04
	*R* ^2^	0.911	0.811
	*SSE*	839.61	873.70
	*χ* ^2^	20.22	13.26
	*ARE*	25.20	18.99
	*HQC*	28.47	28.67

**Table 5:** Literature comparison of sorbents for Pb^2+^ ions and DCF-Na removal.

Sorbent	Sorption Solution	Maximum Sorption Capacity (mg/g) for Pb	Isotherm	Maximum Sorption Capacity (mg/g) for DCF-Na	Isotherm	Reference
Eucalyptus wood biochar	monocomponent	-	-	33.13	Sips	[5]
Hyper-cross-linked polymer (HCP)	multicomponent: Pb, Ni, and Cd	173.10	RSM optimization	-	-	[4]
Magnetic GO/ ZIF-8/γ-AlOOH	monocomponent	-	-	2594.3	Langmuir	[6]
Magnetic amine-functionalized chitosan	monocomponent	-	-	469.48	Langmuir	[23]
Peanut hull-g-methyl methacrylate biopolymer	monocomponent	370.40	Langmuir	-	-	[24]
Activated carbon from cocoa pod husk	monocomponent	-	-	5.67	Experimental	[25]
Activated carbon	monocomponent	-	-	180	Langmuir	[26]
Cross-linked chitosan beads grafted with polyethylenimine	monocomponent	-	-	253.32	Langmuir	[27]
Polyethyleneimine (PEI)-modified chitosan magnetic hydrogel	multicomponent: Pb, Ni, and Cu	100.32	Langmuir	-	-	[28]
Magnetic iron oxide-silica shell nanocomposite	monocomponent	17.1	Langmuir	-	-	[29]
Graphene oxide-silica-chitosan adsorbent	monocomponent	256.41	Langmuir	-	-	[30]
Ordered mesoporous silica nanoparticles of MCM-41 type	monocomponent	22.2	Sips	-	-	[31]
Chemically modified silica monolith	monocomponent	574.71	Langmuir			[32]
IS/(PEI/PMAA)_c_ (r = 1.0)	multicomponent: Pb and DCF-Na	125.68	Langmuir	388.35	Langmuir	This study

**Table 6:** ANOVA summary for the CCD models for the sorption of Pb^2+^ ions and DCF-Na by the IS/(PEI/PMAA)c (r = 1.0) composite sorbent.

	*q_Pb_*					*q_DCF-Na_*				
Source	Sum of Squares	df	Mean Square	*F*-Value	*p*-Value	Sum of Squares	df	Mean Square	*F*-Value	*p*-Value
Model	11,766.72	9	1307.41	856.07	<0.0001	6868.48	3	2289.49	577.59	<0.0001
*A-C0 Pb*	*11,606.63*	*1*	*11,606.63*	*7599.82*	*<0.0001*	*54.77*	*1*	*54.77*	*13.82*	*0.0019*
*B-C0 DCF-Na*	*3.67*	*1*	*3.67*	*2.40*	*0.1521*	*6807.79*	*1*	*6807.79*	*1717.47*	*<0.0001*
*C-T*	*2.47*	*1*	*2.47*	*1.62*	*0.2319*	*5.92*	*1*	*5.92*	*1.49*	*0.2393*
*AB*	*3.05*	*1*	*3.05*	*2.00*	*0.1877*					
*AC*	*5.93*	*1*	*5.93*	*3.89*	*0.0770*					
*BC*	*5.288 × 10^−^* ^3^	*1*	*5.288 × 10^−^* ^3^	*3.462 × 10^−^* ^3^	*0.9542*					
*A* ^2^	*135.62*	*1*	*135.62*	*88.80*	*<0.0001*					
*B* ^2^	*1.72*	*1*	*1.72*	*1.13*	*0.3129*					
*C* ^2^	*0.17*	*1*	*0.17*	*0.11*	*0.7473*					
Residual	15.27	10	1.53			63.42	16	3.96		
*Lack of Fit*	*11.06*	*5*	*2.21*	*2.63*	*0.1561*	*54.75*	*11*	*4.98*	*2.87*	*0.1273*
*Pure Error*	*4.21*	*5*	*0.84*			*8.68*	*5*	*1.74*		
*R* ^2^	0.9987					0.9909				
Adjusted *R*^2^	0.9975					0.9891				
Predicted *R*^2^	0.9920					0.9835				
Adequate precision	123.287					92.668				

**Table 7:** Factor coefficients of the models generated by CCD.

	*q_Pb_*		*q_DCF-Na_*	
Factor	Coefficient Estimate	Standard Error	Coefficient Estimate	Standard Error
Intercept	77.95	0.49	57.20	0.45
A - *C_0, Pb_*	26.93	0.31	1.85	0.50
B - *C_0, DCF-Na_*	0.48	0.31	20.63	0.50
C - *T*	−0.39	0.31	0.61	0.50
AB	0.62	0.44		
AC	−0.86	0.44		
BC	0.026	0.44		
A^2^	−2.32	0.25		
B^2^	−0.26	0.25		
C^2^	0.082	0.25		

## Data Availability

Not applicable.
